# Intracranial Control With Combination BRAF and MEK Inhibitor Therapy in Patients With Metastatic Melanoma

**DOI:** 10.7759/cureus.31838

**Published:** 2022-11-23

**Authors:** Caressa Hui, Yufan (Fred) Wu, Kevin Liu, Navjot Sandhu, Erik Blomain, Michael S Binkley, Melanie H Gephart, Steven D. Chang, Gordon H Li, Sunil A Reddy, Scott G Soltys, Erqi Pollom

**Affiliations:** 1 Radiation Oncology, Stanford University, Stanford, USA; 2 Neurosurgery, Stanford University, Stanford, USA; 3 Oncology, Stanford University, Stanford, USA

**Keywords:** mek inhibition, braf inhibition, melanoma, immunotherapy, stereotactic radiosurgery, radiation therapy

## Abstract

Purpose/Objectives

Combination BRAF (vemurafenib, dabrafenib, or encorafenib) plus MEK (trametinib, cobimetinib, or binimetinib) inhibitor therapy is now widely used in the treatment of metastatic melanoma. However, data for intracranial response to these drugs are limited. We aimed to evaluate the intracranial efficacy of BRAF plus MEK inhibitors in patients with BRAF-mutant melanoma with brain metastases (BM) and to determine patterns of failure of these new agents to inform optimal integration of local intracranial therapy.

Materials and methods

We retrospectively reviewed charts of patients with BRAF-mutant melanoma with metastasis to the brain with at least one untreated brain metastasis at the time of initiation of BRAF plus MEK inhibitors at our institution from 2006 to 2020. We collected per-patient and per-lesion data on demographics, treatment modality, and outcomes. The cumulative incidence of local (LF), distant intracranial (DF), and extracranial failure (EF) were calculated with competing risk analysis with death as a competing risk and censored at the last brain MRI follow-up. LF was calculated on a per-lesion basis while DF and EF were calculated on a per-patient basis. DF was defined as any new intracranial lesions. Overall survival (OS) was analyzed using Kaplan-Meier. Logistic regression was used to identify predictors for LF.

Results

We identified 10 patients with 63 untreated brain metastases. The median age was 50.5 years. The median sum of the diameters of the five largest untreated brain metastases per patient was 20 mm (interquartile range 15-39 mm) and the median diameter for all measurable lesions was 4 mm. Median follow-up time was 9.0 months (range 1.4 months-46.2 months). Median OS was 13.6 months. The one-year cumulative incidence of LF, DF, and EF was 17.1%, 88.6, and 71.4%, respectively. The median time to LF, DF, and EF from the start of BRAF plus MEK inhibitors was 9.0 months, 4.7 months, and 7.0 months, respectively. The larger size of the BM was associated with LF on univariate analysis (odds ratio 1.13 per 1 mm increase in diameter, 95% confidence interval 1.019 to 1.308, p<0.02). Two (20%) patients eventually received stereotactic radiosurgery, and 2 (20%) received whole-brain radiotherapy for intracranial progression.

Conclusion

Although patients with BRAF-mutant melanoma with BM had fair local control on BRAF plus MEK inhibitors, the competing risk of death and distant intracranial and extracranial progression was high. Patients with larger brain metastases may benefit from local therapy.

## Introduction

The treatment paradigm for melanoma has evolved dramatically with the advent of new systemic agents. In addition to novel immune checkpoint inhibitors, BRAF (vemurafenib, dabrafenib, or encorafenib) plus MEK (trametinib, cobimetinib, or binimetinib) inhibitors for patients with BRAF mutants have also shown high rates of rapid response and improvement in overall survival [1-7]. MEK inhibitors allosterically inhibit MEK, leading to cell death, and BRAF inhibitors target BRAF kinase and interfere with the mitogen-activated protein kinase signaling pathway that is responsible for the proliferation of melanoma cells. Studies have shown promising results with anti-CTLA-4 and anti-PD-1 therapy with nivolumab, pembrolizumab, and ipilimumab, with intracranial objective response rates ranging from 16% to 57% with acceptable toxicities [8-11]. Intracranial response rates of patients treated with BRAF monotherapy and BRAF plus MEK inhibitors are also promising, ranging from 18% to 90% with a manageable safety profile [12-15]. However, reported rates of response are widely variable and not durable, and it is important to elucidate how local therapy best fits into this novel treatment paradigm.

There is a paucity of literature surrounding the concurrent use of local intracranial therapies, such as stereotactic radiotherapy (SRS), with BRAF and MEK inhibitors. Small retrospective studies have suggested that SRS with concurrent BRAF monotherapy is associated with excellent local control with no evidence of increased toxicity [16-20]. Our study aims to determine intracranial efficacy, the durability of response, and patterns of failure of BRAF plus MEK inhibitor therapy to inform optimal integration of local intracranial therapy.

This article was previously presented as a poster at the 2021 American Radium Society Annual Meeting on September 29, 2021.

## Materials and methods

Patient population

In this institutional review board-approved retrospective study, we identified patients with metastatic melanoma with a BRAF mutation and brain metastases, treated with BRAF plus MEK inhibitors at our institution from 2006 to 2021. Patients had to have at least one untreated brain lesion at the time of initiation of BRAF plus MEK inhibitor therapy and at least one post-treatment brain MRI.

Patients included in this study either received dabrafenib 150 mg twice daily by mouth with trametinib 2 mg once daily by mouth, encorafenib 450 mg once daily by mouth with binimetinib 45 mg twice daily by mouth, or vemurafenib 960 mg twice daily by mouth with cobimetinib 60 mg once daily by mouth. We did not exclude patients with prior systemic therapies.

Imaging and treatment

The first follow-up brain MRI after baseline imaging was performed between one and three months after patients initiated BRAF plus MEK inhibitor therapy, and follow-up brain imaging is typically performed in three-month intervals if the patient is asymptomatic with regards to their intracranial disease. All sizes of brain metastases were measured by a board-certified radiologist as part of the patient’s clinical care. Multiplanar and multisequence MRI of the brain with a 3T system was performed before and after the administration of gadobenate dimeglumine.

Variables and outcomes

We obtained per-patient and per-lesion data on demographics, treatment, and outcomes. Local failure (LF) was defined as evidence of radiographic progression in the brain lesion. To define the response of the lesions to therapy, we used the modified RECIST (Response Evaluation Criteria in Solid Tumors) 1.1 criteria, which were also used by Tawbi et al. [11,21]. By using a 5 mm threshold for brain lesions, the modified RECIST criteria allowed for the inclusion of additional brain lesions. Comparison between modified RECIST criteria and others such as RANO (Response Assessment in Neuro-Oncology) in evaluating brain metastasis response to systemic therapy show high concordance [22]. However, as stated in the RECIST update and clarification, if the lesion is smaller than 5 mm, but the radiologist believes the lesion can be accurately measured, the actual size was recorded and included in this study [23]. Distant intracranial failure (DF) was defined as evidence of a new lesion in the brain seen on a follow-up MRI. Extracranial failure (EF) was defined as evidence of radiographic progression on positron emission tomography (PET)/CT outside of the brain.

## Results

Patient, tumor, and treatment characteristics

We included 63 untreated brain metastases in 10 patients (Table 1). The median age was 50.5 years old, and 20% were female. The median time to the initiation of BRAF plus MEK inhibitors since the diagnosis of melanoma and diagnosis of brain metastases were 36.9 months and 3.9 months, respectively. The median sum of the diameters of the five largest untreated brain lesions was 20 mm.

**Table 1:** Patient and treatment characteristics IQR = Interquartile Range; LDH = Lactate dehydrogenase; GPA = Graded prognostic assessment; PD1 = Programmed cell death protein 1; CTLA4 = Cytotoxic T-lymphocyte-associated protein 4; WBRT = Whole brain radiation therapy; ECOG = Eastern Cooperative Oncology Group

Characteristic		N (%), unless otherwise specified
Sex		
Female		2 (20%)
Male		8 (80%)
Race		
White		4 (40%)
Unknown		6 (60%)
Age		
<52 years		5 (50%)
≥52 years		5 (50%)
ECOG		
0-1		4 (40%)
2+		1 (10%)
Unknown		5 (50%)
Time since melanoma primary diagnosis (median, IQR)		36.9 months (9.4 months-76.7 months)
Time since diagnosis of brain metastases (median, IQR)		3.9 months (2.9 months-4.8 months)
LDH (median)		303
Unknown		7 (70%)
Steroid dose (prednisone equivalent; median, IQR)		0 mg daily (0-0.75 mg)
Number of untreated brain metastases at time of BRAF/MEK treatment initiation		5 (3-11)
Sum of diameters of 5 largest CNS lesions (mm, IQR)		20 mm (14-44 mm)
GPA score (0-4, IQR)		2 (1.5-2.5)
Presence of extracranial metastasis?		
Yes		9 (90%)
No		1 (10%)
Number of extracranial organs involved (median, IQR)		2 (1.25-4.75)
Number of prior systemic therapies (median, range)		1 (0-2)
Anti-PD1 Only		1 (10%)
Anti-CTLA4 Only		1 (10%)
Both Anti-PD1 and CTLA4		6 (60%)
Other (interferon)		1 (10%)
None		1 (10%)
BRAF/MEK agent used (list below)		
Dabrafenib		8 (80%)
Trametinib		8 (80%)
Vemurafenib		2 (20%)
Binimetinib		1 (10%)
Encorafenib		1 (10%)
Cobimetinib		2 (10%)
Previous local treatment to the brain:		
WBRT		0 (0%)
SRS		8 (80%)
Surgery		4 (40%)
None		2 (20%)
Received concurrent local treatment to brain w BRAF/MEK treatment initiation (within 3 months):		
WBRT		0 (0%)
SRS		3 (30%)
Surgery		1 (10%)
None		6 (60%)
Year of Treatment:		
2012-2016	4 (40%)	
2017-2020	6 (60%)	

At the time of BRAF plus MEK inhibitor initiation, 90% of patients had extracranial metastasis, with a median of 2 extracranial organs involved, and 90% of patients had their primary tumor controlled. Eighty percent (80%) of patients had prior use of anti-PD1 and/or anti-CTLA4 systemic therapies. Two patients had never received previous local treatment for the brain lesions. Three patients had received prior stereotactic radiosurgery while one patient previously underwent surgical resection of a brain lesion. None of the patients had prior whole-brain radiotherapy. Four patients received concurrent (within 3 months) local treatment to the brain with BRAF plus MEK inhibitor therapy. One patient received SRS to a 1.8 cm enhancing recurrent brain lesion along the edge of a resection cavity one month prior to the initiation of BRAF plus MEK inhibitors, and another patient received SRS to a 0.6 cm hemorrhagic brain metastases one month after initiating BRAF plus MEK inhibitors, and finally, one patient received SRS to 2 thalamic brain lesions one week prior to starting BRAF plus MEK inhibitors, measuring 1.0 cm and 0.7 cm. This same patient also underwent surgical resection of a 1.7 cm hemorrhagic brain lesion 1 week prior to starting BRAF plus MEK inhibitors. The median size of the lesions treated with concurrent SRS or surgery was 1 cm.

Overall survival, progression-free survival, local failure, distant intracranial failure, and extracranial failure

**Figure 1:**
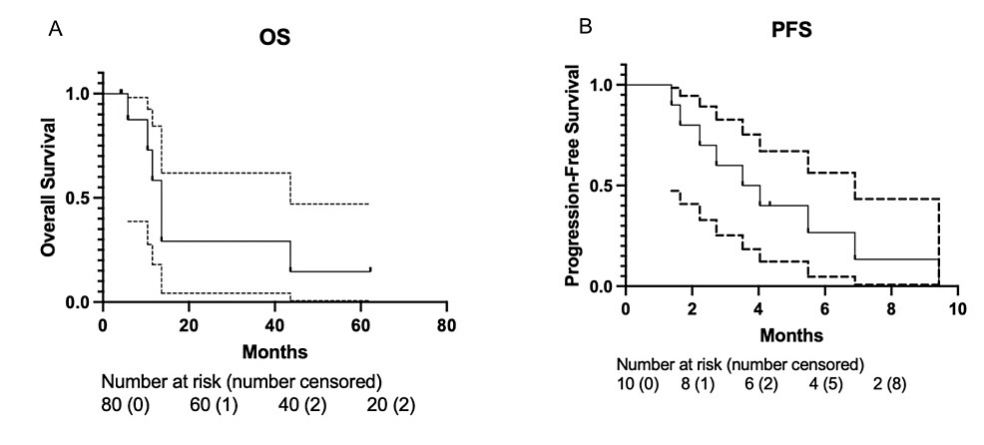
A: Overall survival; B: Progression-free survival

**Figure 2:**
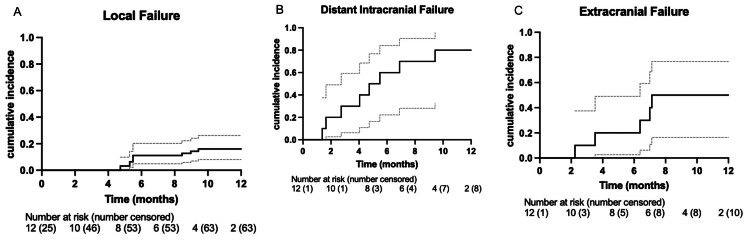
A: 1-year local failure cumulative incidence per lesion; B: 1-year distant intracranial failure cumulative incidence; C: 1-year extracranial failure cumulative incidence

The median overall survival of the entire cohort was 13.6 months after the initiation of BRAF plus MEK inhibitors (Figure 1A). The initial intracranial response rate was 60%. The remaining 40% of patients had progression of their intracranial disease at the first MRI follow-up. The median time to the first MRI follow-up was two months. Median progression-free survival (PFS) was 3.8 months (95% CI 0.596-0.995, Figure 1B). Of those who progressed, the median times to LF, EF, and DF were nine months (95% CI 0.124-0.322, Figure 2A), 4.7 months (95% CI 0.237-0.763, Figure 2B), and 7.0 months (95% CI 0.596-0.995, Figure 2C), respectively. The one-year cumulative incidence of LF, EF, and DF was 17.1%, 71.4%, and 88.6%, respectively. Two (20%) patients eventually received stereotactic radiosurgery (SRS) for growing lesions, and two (20%) received whole-brain radiotherapy (WBRT) for intracranial progression. Of the 63 lesions included in the study, 7 lesions (11.1%) were eventually treated with salvage SRS. The median duration of BRAF plus MEK inhibitors in the entire cohort was 6.6 months.

Factors associated with local failure

A larger size of the brain metastasis was associated with LF on univariate analysis (odds ratio 1.13 per 1 mm increase in diameter, 95% confidence interval 1.019 to 1.308, p<0.02). Forty-two percent of lesions with a maximum diameter greater than 1 cm in size progressed and experienced local failure. Thirty-six percent of lesions greater than 5 mm in size progressed, whereas only 13% of lesions 5 mm or smaller experienced an LF. Age, Eastern Cooperative Oncology Group (ECOG), number of extracranial organs involved, and the sum of the largest five diameters of brain metastasis were not associated with LF.

Toxicities

Six patients (60%) experienced significant toxicities with BRAF plus MEK inhibitors that required switching to another BRAF plus MEK inhibitor (1 patient), treatment breaks or decreased dose (4 patients), or stopping therapy completely (1 patient).

## Discussion

Historically, therapeutic treatment for patients with melanoma brain metastases was limited to surgery or radiation therapy. The introduction of BRAF and MEK inhibitors profoundly impacts the treatment paradigm of patients with metastatic melanoma. With the improved intracranial response rates compared to prior agents, we sought to elucidate the role of local therapies, such as SRS, in the context of BRAF and MEK therapy.

**Figure 3:**
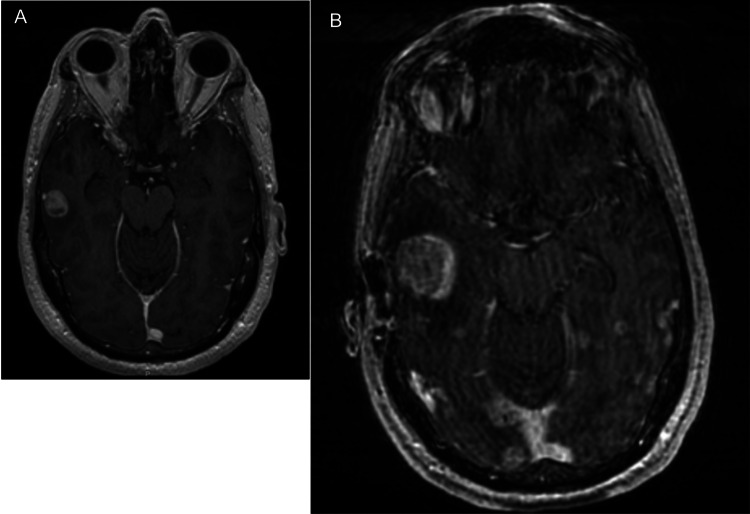
A: Patient’s T1-weighted MRI at the initiation of BRAF plus MEK inhibitors, showing a 1.5 cm brain lesion; B: Patient’s T1-weighted MRI six months after initiation of BRAF plus MEK inhibitors, showing the same lesion that now measures 3 cm The patient is symptomatic with seizures.

We found that intracranial response rates to BRAF and MEK therapy were high (60%), as other studies have found [12-15]. However, the duration of intracranial control was short-lived, with a median time to local and distant intracranial failure of only nine and seven months, respectively. Given the competing risk of death and distant intracranial and extracranial progression in these patients, local therapies, such as SRS, will not improve outcomes at the time of initiation of BRAF and MEK therapy for most patients. However, we found that larger tumors were associated with worse local control on BRAF and MEK therapy, suggesting that upfront SRS may be considered for larger tumors to decrease the risk of symptomatic progression. Other studies have also reported poorer local control with larger size of brain lesions [24-26]. A patient who was included in our study initially had a 1.5 cm brain metastasis; local treatment was deferred and BRAF plus MEK inhibitors were started (Figure 3A). Six months later, the patient presented to the emergency room with seizures, and MRI showed a doubling in diameter of the tumor to 3 cm (Figure 3B). Thus, in addition to the larger size at presentation, other clinical factors, such as location and symptoms associated with brain metastases, should also be considered in the individualization of treatment for these patients. Although there have been no published studies that show an improvement in survival with the addition of SRS for melanoma brain metastases in patients on BRAF plus MEK inhibitors, it may be reasonable to offer upfront SRS if the lesions are larger, to prevent worsening neurological symptoms, with no significant increases in toxicities.

**Figure 4:**
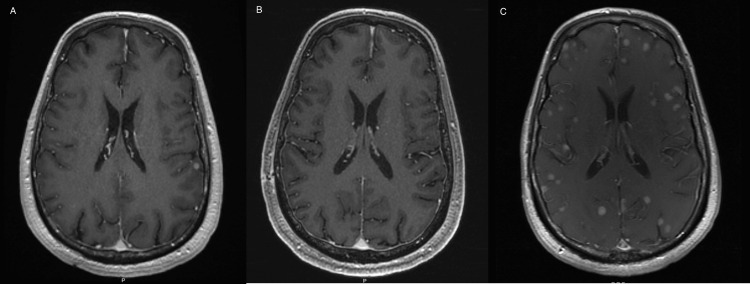
A: Patient’s T1-weighted MRI at the initiation of BRAF plus MEK inhibitors, showing multiple brain lesions; B: Patient’s T1-weighted MRI one month after initiation of BRAF plus MEK inhibitors, showing resolution of all previously visualized brain lesions; C: Patient’s T1-weighted MRI five months after initiation of BRAF plus MEK inhibitors with innumerable new brain lesions

In addition to the improvement of neurological symptoms and high local tumor control rates with radiation therapy, there may be synergy between BRAF plus MEK inhibitors and radiation. A preclinical study by Sambade et al. treated melanoma cell lines with radiation therapy and the B-RAF inhibitor PLX-4032 and found enhanced inhibition of colony formation and invasion, and additionally radio-sensitized cells through an increase in G1 cell cycle arrest [27]. Furthermore, radiation therapy could cause a transient disruption of the blood-brain barrier, causing increased permeability, subsequently allowing increased drug uptake [28,29]. Finally, although previous studies have detailed impressive CNS responses with BRAF plus MEK inhibitors alone, the duration of these intracranial responses may be prolonged with the synergistic effects of radiation therapy. For example, one patient that was reviewed in our study had not undergone upfront radiation therapy for his asymptomatic brain metastases, was started on BRAF plus MEK inhibitors, and experienced a dramatic response to BRAF plus MEK inhibition that was seen on the first follow-up brain MRI one month after initiation of systemic therapy (Figures 4A-4B). However, a surveillance MRI done subsequently five months after the initiation of systemic therapy showed innumerable brain metastases (Figure 4C). Although many of the exact mechanisms are unknown, the one-year local brain control in patients treated with BRAF inhibitors and concurrent radiation therapy is excellent in published retrospective studies, ranging from 75%-92%, suggesting a potential synergistic effect that translates into a clinically meaningful benefit for patients [16-20].

Reports evaluating the safety of radiotherapy and BRAF inhibitors are limited. For example, a small retrospective study by Narayana et. al included 12 patients with BRAF mutant melanoma with brain metastases who underwent radiation therapy prior to or during vemurafenib and found that two patients had developed steroid dependence due to intracranial edema and one patient experienced radiation necrosis. However, they found that neurologic symptoms were improved in 64% of their patients and 75% of the brain metastases treated had a radiographic response and concluded that these patients have a favorable response to vemurafenib and radiation therapy with acceptable morbidity [16]. In our study, we did not find any significant radiation-related toxicities in the four patients that eventually underwent SRS or WBRT, or in patients who received concurrent local therapy with BRAF and MEK inhibitors. In addition, of the patients who experienced toxicities with BRAF plus MEK inhibitors that required treatment breaks or stopping therapy, none were related to the use of radiation.

This single-institution retrospective study has several inherent limitations. Our cohort of patients is heterogenous, treated with various local treatment modalities spanning over a decade. Although our retrospective study included a small number of patients, we included per-lesional analysis and per-lesional outcomes and tracked the response of 63 lesions over a total of 70 MRI scans for robust local control outcomes. Existing data are particularly lacking in terms of per-lesional outcomes, with some studies reporting excellent intracranial responses but only on a per-patient basis.

Additional research using prospective or large retrospective cohorts is necessary to validate whether the use of local radiation therapy in addition to BRAF plus MEK inhibitors improves outcomes in patients with BRAF mutant melanoma with brain metastases. Furthermore, with the increasing integration of other systemic therapies, such as immunotherapies, which can improve the durability of extracranial disease control, local control of brain metastases may become more important moving forward. There are currently several ongoing studies that are actively recruiting and investigating the activity of BRAF plus MEK inhibitors plus radiation therapy. The EBRAIN-MEL study (NCT03898908) evaluates the outcomes for patients on encorafenib and binimetinib plus either WBRT or SRS [30]. The RadioCoBRIM trial (NCT03430947) studies outcomes in patients started on vemurafenib plus cobimetinib after receiving SRS [31]. The results of these studies will hopefully guide the optimal integration of local intracranial therapy in the context of these novel target therapies.

## Conclusions

Although patients with BRAF-mutant melanoma with brain metastasis had fair local control on BRAF plus MEK inhibitors, the competing risk of death and distant intracranial and extracranial progression in our institutional experience was high. Patients with larger brain metastases may benefit from local therapy to prevent symptomatic intracranial progression. Further studies should be done to further evaluate the optimal role and timing of local therapy in adjunct to systemic therapy with BRAF plus MEK inhibitors.
